# Dermoscopic features of sarcoidosis

**DOI:** 10.11604/pamj.2014.18.111.4613

**Published:** 2014-06-04

**Authors:** Iman Hadj, Fatima Zahra Mernissi

**Affiliations:** 1 Service de dermatologie, CHU Hassan II, Fès, Maroc

**Keywords:** Dermoscopy, sarcoidosis, granulomas, inflammatory disease

## Image in medicine

Sarcoidosis is a multisystemic, inflammatory disease of unknown aetiology that is characterized by noncaseating granulomas. While numerous organs may be involved, the lungs, lymph nodes, and skin are most commonly affected. Therefore, in recent years dermoscopy is gaining appreciation in the era of inflammatory skin diseases such sarcoidosis, it allows better visualization of dermal vascular structures and color variations. Sarcoidosis is dermoscopically characterized by translucent yellow to orange globular-like or structureless areas, possibly corresponding to the well defined sarcoid granulomas, and linear or branching vessels. We report the case of a 45-year-old women, hospitalized for an asymptomatic erythematous plaque of the noose lasting for 5 mounths. The patient did not report development of similar lesions in the past and his medical history was otherwise unremarkable. Clinical examination revealed a 4x3 cm infiltrated erythematosquamous plaque affecting the nasal raile. Dermoscopically, the lesion exhibited Orange-yellowish patch with branching vessels. Histopathology examination was in favour of sarcoidosis, all examination in search of extra cutaneous involvement was negative. The patient was treated with topical corticosteroid.

**Figure 1:**
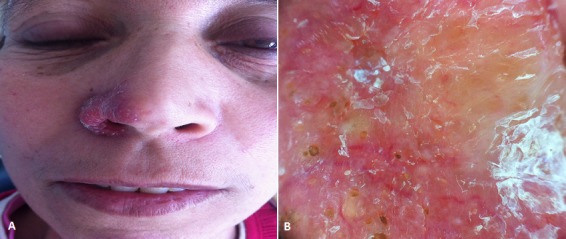
A) Erythemato-squamous plaque of the nose; B) Orange-yellowish patch with branching vessels

